# The dark side of personality functioning: associations between antisocial cognitions, personality functioning (AMPD), empathy and mentalisation

**DOI:** 10.3389/fpsyt.2024.1377177

**Published:** 2024-05-28

**Authors:** Luna Rabl, Jeff Maerz, Roberto Viviani, Karin Labek

**Affiliations:** ^1^ Institute of Psychology, University of Innsbruck, Innsbruck, Austria; ^2^ Dept. of Psychiatry and Psychotherapy III, University of Ulm, Ulm, Germany

**Keywords:** dark personality, personality functioning, empathy, mentalization, criterion A, scrambled sentences task, sst

## Abstract

**Introduction:**

With the introduction of the new psychiatric diagnostic manuals, personality functioning has gained new prominence. Several studies have reported consistent findings that individual showing high levels of antisocial features are associated with alterations in interpersonal functioning domains such as empathy and mentalisation. The focus of the current study (*N* = 198) is to examine antisocial cognitions, as measured by the Scrambled Sentences Task (SST), and to what extent this approach can help to better understand the relationship between antisocial traits and personality functioning/empathy.

**Method:**

We implemented a hypothesis-driven approach using logistic regression and a data-driven approach using machine learning to examine distinct but related measures of personality functioning as predictors of antisocial cognitions.

**Results:**

Antisocial cognitions were associated with low interpersonal functioning as expected, but only when not adjusting for antisocial traits, which accounted for almost all the association. The data-driven analysis revealed that individual items assessing empathic concern in personality functioning scales (as opposed to the whole scores) explained low antisocial cognitions even when adjusting for antisocial traits.

**Discussion:**

Antisocial cognitions appear to be associated to two distinct traits, the antisocial and a specific type of personality functioning. This finding is discussed in terms of the possible distinction between two motivational forces: to harm others/prioritize one’s advantage, and to help suffering others.

## 1 Introduction

The current diagnostic manuals DSM-5/DSM-5-TR (fifth version of the Diagnostic and Statistical Manual of Mental Disorders/fifth version of the Diagnostic and Statistical Manual of Mental Disorders Text Revision) (1) and the eleventh revision of the International Classification of Diseases (ICD-11) (2) offer a dimensional approach to diagnosing personality disorders. Rather than psychopathological symptoms and categorical entities, this approach characterizes disordered personality in a space defined by five trait dimensions and a severity dimension (3). We focus here on the additional characterization of the severity of the disorder (criterion A), defining pathology as one end of a continuous spectrum that includes healthy individuals in the general population at the other end. The general diagnostic criterion for personality disorders (PD) severity in current manuals is now referred to as ‘personality functioning’ in the Alternative Model of Personality Disorders (AMPD of the DSM-5 and DSM-5-TR (criterion A, section III) (1, 4, 5) and in the ICD-11 (2, 6–10). Personality functioning is expected to be useful not only in clinical populations but also in healthy individuals to understand or prevent the development of difficult interpersonal behaviour.

Both the AMPD and ICD-11 manuals use a self-functioning (e.g., identity, self-direction) and an interpersonal-functioning domain (e.g., empathy, intimacy) to describe the severity of impairment in personality functioning. The basic notion behind this approach is that the coexistence of various PD symptoms is caused by a shared underlying core of impaired intrapsychic functioning (4, 11), which is characterized by these impairments. From a clinical point of view, the study of personality functioning has been approached by investigating the extent to which it epitomized by or overlaps with the symptoms of borderline personality disorder (BPD) (12–15) or narcissism (11, 16). Because BPD and narcissistic patients (14, 17, 18) are commonly seen in the clinic, they may have drawn most attention in the clinical characterization of personality functioning.

Recently, however, attention has also been given to the relationship between impaired personality functioning and antisocial personality traits, motivated by the notion that empathy especially may be impaired in antisocial individuals (19–22). The AMPD offers a description of the personality functioning of the antisocial personality as distorted mental representations of self and others. This results in impairments in self-functioning, characterized by egocentrism and the absence of internal prosocial standards and interpersonal dysfunction, as described by a lack of concern for others and of remorse, exploitativeness, the use of deception, coercion, dominance, and intimidation (1). Personality functioning in the AMPD/ICD11 and the related mentalization concept (20, 23) reflect the quality of representations as internal mental states of self and others, which is considered fundamental for building healthy social relationships and enabling adaptation to the social environment (24–26). Individuals who engage in antisocial behaviour have consistently demonstrated impairments in central social capacities, such as empathy and mentalization resulting in problematic social functioning in general (19, 20, 27–30), as well as impulsive and destructive social interactions that violate other people and social norms (26, 31). Hence, the issue arises of the extent to which the low empathic capacities of low personality functioning are coextensive with antisocial tendencies, or whether the construct of empathy may be too heterogenous and contain traits that are differentially characteristic of antisocial personality.

Alongside personality traits associated with antisocial behaviour also included in the clinical diagnostic manuals (e.g. antagonism in the DSM-5), there are also subclinical traits that have emerged from this clinical tradition (32). For example, classic texts have described psychopathic personalities as leading their life unrecognized in society, contributing to our current understanding of psychopathy (33). Also following a dimensional approach to the description of personality traits, these subclinical traits have been described for decades outside the tradition of clinical nosography in the scientific context under the term of “dark personality” (dark P) (34–38). Dark P is used to refer to people with high levels of so-called dark traits, covering a collection of related but theoretically distinct antisocial/dissocial personality constructs. The most commonly studied dark personality traits are psychopathy, Machiavellianism and narcissism, known as the dark triad (34, 39–41), recently extended to include sadism (dark tetrad) (39). Also in this research tradition, some researchers have argued that lack of empathy as specific feature of dark traits may be considered the core of dark personality (41, 42), whereby sub-traits of the dark personality are not only defined by this common core, but contain further individual characteristics, as shown in the example of callousness, a lack of guilt and a restricted affect. Persons high in dark personality strive to maximise their own utility, disregarding, accepting or even provoking disutility to others (43). Like personality functioning, the construct of dark personality is known to capture subclinical traits as well (32, 44–47).

Recently, an instrument originally developed to assess depressogenic cognitions (Scrambled Sentences Task, SST) (48–52) has been adapted to assess the preference to entertain antisocial cognitions. This instrument has provided empirical evidence that people higher in dark personality traits are more prone to activate antisocial cognition belonging to schemas that are in line with the motivation to maximise one’s own utility, the motivation to harm others, and accompanying justificatory beliefs. Those beliefs are often manifested in a particular type of worldview, such as that the world is a dangerous place and every individual needs to look out for themselves (43, 53, 54).

The aim of this study was to use this instrument (Scrambled Sentences Task for Antisocial Cognitions) to assess individual differences in the tendency to activate antisocial (or, inversely, prosocial) cognitions to determine to what extent this approach can contribute to understanding the relationship between antisocial traits on the one hand and personality functioning on the other. Within personality functioning, we focused on those aspects that may be most affected in antisocial personality, i.e. empathy and mentalization capabilities. In previous studies, we have reported on the association between antisocial traits and antisocial cognitions, as assessed by the SST. Here, we hypothesized that antisocial cognitions would also be higher in individuals with low empathy and low mentalization, If so, the question arises of whether these two personality traits exert separate influences on antisocial cognitions, given that antisocial traits and low mentalization are themselves associated in population samples. Thus, any association between antisocial cognitions and low mentalization could simply be due to low mentalization being confounded with antisocial tendencies. After verifying that low empathy and mentalization were associated with higher antisocial traits, our strategy was to investigate the extent to which these two different sets of traits were differentially predictive of antisocial cognitions, assessed with the SST.

### 1.1 Analysis strategy of the current study

The analysis was conducted with two approaches. In the first approach, we estimated hypothesis-driven logistic regression models to verify the expected association between the tendency to entertain antisocial cognitions and low personality functioning, without and then with adjustment for dark personality scores. Our intent was to explore the extent to which aspects of personality functioning, and especially capacity for empathy, were associated with antisocial cognition over and above the association explained by antisocial personality traits, and exclude antisocial traits as a confounder of the association with personality functioning. These models revealed that the association between antisocial cognitions and most measures of personality functioning was modest, and that this association disappeared almost entirely after adjusting for dark personality scores. These models also revealed the existence in our sample of individuals with relatively high scores in empathy who nevertheless showed a considerable propensity towards antisocial cognitions.

In the second explorative approach, we employed a data-driven machine learning technique (conditional importance of predictors in random forest models) (55, 56) to identify individual items of the personality functioning scales that were predictive of high antisocial cognitions. The difference with the logistic regression of the first approach is that all items of the personality functioning scales were used as individual variables in the model to predict the rate of antisocial cognitions, instead of the overall scores. Our intent here was to inquire if the modest associations uncovered by the first phase of the analysis may have been due to specific aspects of personality functioning that were diluted in the overall personality functioning scales (where prosocial or antisocial features are not a primary target), resulting in only modest associations after adjusting for dark personality scores. If the constructs assessed by these scales were heterogeneous with respect of their association with antisocial traits, we may expect the capacity of this alternative model to predict antisocial cognitions to increase, as the model may select the items that are differentially capable of predicting these cognitions. The conditional importance approach was chosen as it has been shown in systematic reviews to be particularly effective in identifying important predictors in models with many variables (57). In the last step, we estimated a new model of the tendency to entertain antisocial cognitions where the original personality functioning scales were replaced by the items identified with the machine learning approach. We found that the association persisted even after adjusting for dark personality scores, in contrast to the original scales of the first approach, suggesting that specific aspects of personality functioning, but not the original scale constructs, may be independently contribute to the propensity towards antisocial cognitions.

## 2 Methods

### 2.1 Sampling and study design

The study was designed using LimeSurvey software to collect the data from the questionnaires. The SST was conducted by in-house software coding a web application and was accessed through a link (53, 58, 59). Software used including the items of the SST for antisocial cognitions (in German) is available on request from Luna Rabl.

We distributed the link to the survey via the mail server of the University of Innsbruck. Participants who were also studying psychology in Innsbruck received 0.75 participant hours as an incentive. In total we were able to recruit *N* = 272 participants completing the rating scale data. Two participants were excluded because of age less than 18 and 10 because they participated twice in the experiment. In the SST data, we excluded participants who did not participate in the SST (*N* = 63) or took less than 2 seconds on average to complete each SST item (12 participants). After combining the SST data and the behavioural data, we obtained a dataset of 198 participants who completed all questionnaires and the SST. The mean age was 23.27 years with a standard deviation of 5.95. Gender distribution showed that more females (*N* = 137) participated in this study than males (*N* = 60) and non-binary (*N* = 1). Most participants were students (*N* = 184) with high-school diploma as highest education level (*N* = 165), with a minority having completed university education (*N* = 25).

### 2.2 Instruments

The development and validation of the SST for antisocial cognition has been previously described (53). Personality functioning was assessed with different but related measures to capture overlapping aspects of personality functioning, and of mentalization capacities in particular, and to increase the external validity of conclusions about the relationship between personality functioning and antisocial cognitions. Antisocial tendencies were assessed with specific scales applicable to the general population, but also including an evaluation of sadistic tendencies.

#### 2.2.1 Scrambled sentences task for antisocial cognitions

The SST for antisocial cognitions (53) was developed according to the scheme of pre-existing SST (48, 49). The SST contains six jumbled words from which two different sentences can be formed. These sentences differ only through one word (the target) at the end of the sentence (49). The targets refer to a specific word in the sentence (called anchor) and represent two different schemata, usually a positive and a negative schema. For example, in the sentence *the future looks very bright/dismal* “bright” represents the positive schema und “dismal” is the negative schema, “future” on the other hand is the anchor (49, 50, 60). Originally the SST was conducted in paper-pencil-format but can nowadays also be conducted online (48–50, 60). The convergent validity and reliability of the instrument are rated to be good (52).

The SST for antisocial cognitions contains in total 44 items, which can be divided into two subgroups *Justifications* (21 items) and *Harm* (23 items). Harm sentences containing harm to others and/or the maximisation of one’s own utility (e.g. *little Tim gets a spanking/praise*). Justifying sentences containing believes that can help to justify the actions descripted in the Harm subcategory (e.g. *Narratives about honesty are fictional/inspirational*). In the SST for antisocial cognitions the negative target represents an antisocial schema, and the positive target represents a more prosocial schema. In this study the participants had 8 seconds for each sentence to form them in their mind and select the preferred target.

#### 2.2.2 Personality functioning

For measuring the personality functioning, an originally 80 item self-report scale (61) and a short form in English was developed (62). In this study, the German version of the Level of Personality Functioning Scale-Brief Form 2.0 (LPFS-BF) was used (63). The LPFS-BF contains 12 items and can be divided into two subscales, interpersonal functioning (e.g. *My relationships and friendships never last long*) and self-functioning (e.g. *I often do not know who I really am*) each containing six items that must be answered on a 4-point Likert scale (1 (*completely untrue*) to 4 (*completely true*)). The reliability of the interpersonal functioning subscale ranges from .83 to .87. The reliability of the self-functioning subscale is even higher, ranging from .86 to .90 and the total score has a reliability between .93 and .94 (63). In this study we found a reliability of .79 for the total score of personality functioning, which can be considered as good.

#### 2.2.3 Empathy

The Saarbrücker Persönlichkeits-Fragebogen (SPF) was used to measure empathy (64). This scale is a further development and German translation of the Interpersonal Reactivity Index (IRI) (65). In total, the SPF contains 16 items that capture four constructs (perspective taking, fantasy, empathic concern and personal distress) of empathy (e.g. I often have tender, concerned feelings for people less fortunate than me.). Each item can be answered from 1 (*never*) to 5 (*ever*). Cronbach’s alpha for empathic concern and perspective taking is .71, fantasy has a reliability of .74 and α for personal distress is .66 (64). In our study we found an acceptable reliability for empathic concern (.62) and perspective taking (.68), as well as a good one for fantasy (.76) and personal distress (.75).

#### 2.2.4 MZQ

The Mentalization Questionnaire (MZQ) was used to measure mentalisation (66). The scale contains a total of 15 items (*Sometimes I only become aware of my feelings in retrospect*), which are scored by taking the mean. Each item can be answered on a 5-point Likert scale ranging from 1 (*Totally disagree*) to 5 (*Totally agree*). A Cronbach’s alpha of .81 was found for the total mean score (66). In this study, the total mentalization score yielded an α coefficient of .84, indicating good internal consistency.

#### 2.2.5 Dark personality

We used two different scales to measure dark personality. The Short Dark Triad (SD3) measures psychopathy, Machiavellianism and narcissism (47). To complement the dark tetrad we added the Assessment of Sadistic Personality (ASP) to capture sadism (40). Each of these four subscales contains 9 items (e.g. of the ASP: *I have made fun of people so that they know I am in control.*; e.g. of the SD3: *Most people can be manipulated.*), that can be answered on a 5-point Likert scale (1 = *strongly disagree*, 5 = *strongly agree*). A Cronbach’s alpha of .83 was found for the ASP (40). In the SD3, reliability for psychopathy ranged from .72 to .73, for Machiavellianism from .74 to .76 and for narcissism from .68 to .78 (47). In our study, we found an acceptable to good reliability of .76 (Machiavellianism), .72 (narcissism), .69 (psychopathy) and .82 (sadism).

### 2.3 Statistical analysis

The pre-processing (e.g. exclusion of participants, standardisation) of the data was conducted using the software SPSS (version IBM SPSS Statistics 24) and RStudio (version R 4.2.1). Subsequent statistical analyses were exclusively conducted in RStudio (version R 4.2.1) using the ‘dplyr’ package for data manipulation.

*Repeated Measurement Mixed-effects Logistic Regression (RM-LG).* The package *lme4* was used to analyse the data obtained from the SST (answers were coded dichotomously, with a higher score indicating a higher tendency to choose the prosocial target.) using mixed-effects logistic regression (67). Results within models were controlled for gender and age, subjects and the SST sentences were modelled as random effects to account for repeated measurements. All models could be fitted according to the convergence diagnostic of the *lme4* package.

*Random Forest Regression Conditional Variable Importance (RF-CVI):* A notable advantage of machine learning algorithms, such as random forests, lies in their ability to provide a hierarchical measure of the importance of each predictive variable. In the Conditional Variable Importance approach, the importance value is computed via conditional permutation of the predictors, based on the decrease in predictive accuracy due to the permutation. In our implementation, the random forest regression model was executed using the *‘party’* package (55, 56). For the current study, we opted for the conditional permutation importance variable approach (55, 56) over the original permutation importance method (68). This choice was motivated by concerns that the original method might be susceptible to spurious correlations, leading to an overestimation of the importance of correlated variables. The RF-CVI method has been described in the literature to provide more reliable results of the true impact of each predictor compared to the original marginal approach (55, 56). Additionally, it enables the consideration of potential confounding factors by conducting the permutation importance assessment only conditional on the values of other features that are correlated with the feature under investigation.

In our study RF-CVI models were built with 43 features (single items of all personality functioning questionnaires) to predict the ratio of prosocial sentence choices in each individual. To verify the replicability of the estimates of variable importance, the original dataset was first randomly split between two datasets of equal size (*N* = 99), on which the RF-CVI analysis was computed independently. These two analyses were conducted with parameters selected via a grid search (1000 trees, 20 predictive features per tree, tree depth 14). The fit of each model was repeated 88 times with different values from the random number generator, using the same seed for the two datasets, and averaging the results within each dataset. We then visualized the correlation between the conditional importance of each variable as computed in the two datasets (shown in Figures in the Results section). After verifying the replicability, we used the whole dataset to compute final conditional importance of all items. A supplementary RF-CVI analysis was computed including all items of all scales (personality as well as dark P), to verify the robustness of the selection of personality items to predict cognitions. Further information on the parameters we selected in RF-CVI may be found in the **Supplementary Material**.

**Figure 1:**
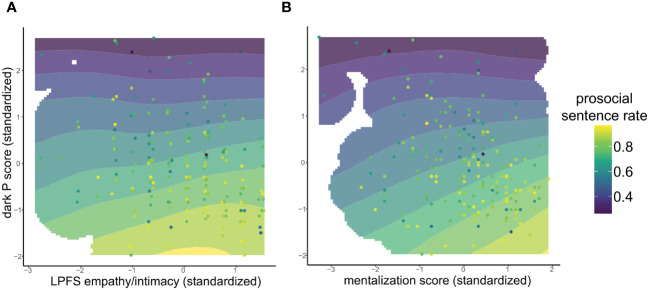
This figure shows the probability of forming a prosocial sentence as a function of dark P scores and the personality scales of empathy and intimacy **(A)** and mentalization **(B)**. The points are the prosocial sentences ratio of individual participants. Blank regions in the plot are due to lack of observations in that score range.

**Figure 2:**
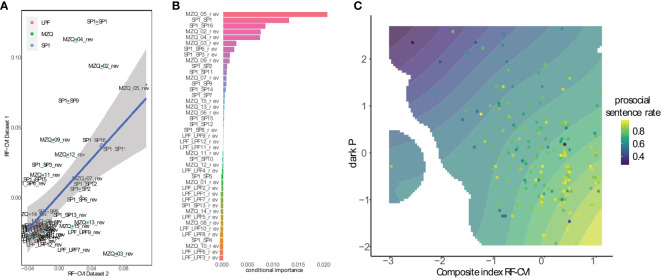
**(A)** scatterplot of random forest regression conditional variable importance (RF-CVI) scores between Dataset 1 and Dataset 2. The association, shown with 95% confidence intervals, assessed the extent of replicability of the variable importance analysis between these two datasets selected at random. **(B)** relative values of the RF-CVI computed on the entire dataset. The variables at the top are best at predicting the prosocial sentence rate across individuals. The values on the x axis are unitless and should be interpreted as providing the relative capacity of variables to predict the outcome. Small negative values at the bottom of the ranking hierarchy are common in this kind of analysis and indicate that the variables provide little or no contribution to prediction given the other variables in the model. **(C)** fitted probability of forming a prosocial sentence as a function of dark P scores and the combined first two variables identified by the RF-CVI analysis. The points are the prosocial sentences ratio of individual participants. Blank regions in the density are due to lack of observations in that score range.

*Illustrations and Plots:* Plots were created with the package ggplot2, version 3.3.6 (69, 70). The fitted surface of ratio of prosocial sentences of **Figures 1** and **2** were created with the function brm of the brms package (71). Individual rates of prosocial sentence selection was modelled in a mixed-effects logistic regression with subjects and SST sentences as random effects, thin-plate splines to model the surface, with age and gender as confounding covariates. The package brm implements a Bayesian approach, which estimates the degree of smoothing from the data through the estimated variance parameter of the coefficients of thin-plate splines modelled as a random effect (72).

## 3 Results

The analysis in the upcoming section is structured as follows. After reporting the raw correlations between the variables in the study, we model the predictive value of personality functioning measurements, both with and without adjusting for dark P, in prosocial sentence choice with repeated-measurements logistic regression. Subsequently, an exploratory data-driven approach was used to identify the most important items in the personality function scales that may be predictive of prosocial sentence selection. To this end, we computed the conditional variable importance method in a random forest model (55, 56). This method, which was shown in comparative analyses to outperform other approaches for variable identification (73), delivers values that may be used to rank the relative importance of individual variables in predicting the outcome. Finally, relevant variables found in the random forest model were included into the repeated-measurements regressions models to determine whether they reveal stronger effects on the propensity for sentence choice compared to the models that include the total scales.

SST responses were coded such that the selection of a prosocial sentence was scored positively. Hence, a negative coefficient of dark P means that individuals with higher scores were making less prosocial (more antisocial) sentences. To facilitate interpretation of coefficients of personality functioning scales, scores on the LPFS (personality functioning total score and for the interpersonal subscale), MZQ (mentalization), and IRI empathy concern, fantasy and perspective taking scales were reversed in the direction to indicate consistently that higher scores corresponded to higher abilities (in the case of personal distress a higher score means less distress), irrespective of whether they originally indicated lower functioning, or lower empathy. Thus, a positive coefficient in these scales indicates a higher tendency to select prosocial sentences. In the SST used here, where the alternative sentences that could be selected were prosocial and antisocial, a higher tendency to select prosocial sentences is synonymous with a lower tendency to select antisocial sentences, and vice versa.

### 3.1 Correlation between variables

The correlations between the variables used in the study are shown in **Table 1**. They revealed strong positive associations between the Level of Personality Functioning (LPFS, both total and subscales self/other) and mentalization (MZQ), in line with existing literature (23, 44, 59). Additionally, LPFS and MZQ showed significant correlations with personal distress, suggesting a possible link to an individual’s general capacity or vulnerability, particularly in distressing situations such as the misfortune of others.

**Table 1:** Correlation table between the variables used in the study.

		M	SD	1	2	3	4	5	6	7	8	9	10
1	Gender	F=137											
2	Age	23.27	5.95	0.00									
3	LPFS total	1.96	0.47	0.05	-0.20								
4	LPFS self	2.23	0.63	0.14	-0.27*	0.90*							
5	LPFS other	1.70	0.45	-0.09	-0.04	0.80*	0.47*						
6	MZQ total	3.60	0.63	-0.03	0.18	-0.76*	-0.67*	-0.63*					
7	IRI empathic concern	3.86	0.57	0.38*	0.02	0.10	0.17	-0.04	-0.09				
8	IRI fantasy	3.70	0.75	0.11	-0.12	0.14	0.18	0.04	-0.06	0.39*			
9	IRI personal distress	2.80	0.76	0.30*	-0.18	0.52*	0.51*	0.37*	-0.48*	0.27*	0.21		
10	IRI perspective taking	3.85	0.60	0.10	-0.08	-0.11	-0.02	-0.20	-0.01	0.19	0.20	-0.04	
11	DP	2.19	0.46	-0.38*	-0.02	0.19	0.07	0.29*	-0.31*	-0.34*	-0.07	-0.15	-0.19

M, mean; SD, standard deviation; DP, total score of the dark personality (narcissism, Machiavellianism, psychopathy and sadism), * p <.05 after Bonferroni correction, n = 198.

Regarding the dark P scale, the LPFS other-subscale, the MZQ scale and IRI empathic concern scale showed significant correlations. The findings suggest that individuals with higher scores on antisociality traits tend to have lower scores on personality functioning, indicating a potential impairment in specific domains.

The correlations with gender also suggest that women experienced higher levels of distress, but also showed greater empathic concern and less antisocial tendencies.

### 3.2 Predictive value of personality on prosocial sentence choice

In the SST, participants showed the expected general tendency (*z* = 9.87; *p* <.001) to form prosocial sentences, with no effects for gender and age. We examined the associations between the choice of prosocial SST sentences and the personality functioning total score (Model 1.0) and its self- and interpersonal sub-scores (Model 2.0), the mentalization total score (Model 3.0) and the IRI scales (Model 4.0). We expected that, after adjusting for age and gender, participants with high scores on the functioning scales would be more likely to select prosocial sentences.

Starting with the personality functioning total score (LPFS, Model 1.0 in **Table 2**) the analysis of the expected relationship between the rates of prosocial sentences revealed a non-significant association, with a p-value of 0.08. In contrast, the empathy and intimacy subscale demonstrated a significant association (Model 2.0, LPFS interp.).

**Table 2:** Comparison of models.

Model	Predictor	Odds ratio	z	p	Model	Predictor	Odds ratio	z	p
Model 1.0	LPFS total	0.90 (0.85-0.96)	−1.75	0.081	Model 1.1	LPFS total	0.97 (0.91-1.02)	−0.60	0.546
						Dark P	0.71 (0.67-0.76)	−5.53	< 0.001
Model 2.0	LPFS self	0.99 (0.92-1.06)	−0.14	0.889	Model 2.1	LPFS self	0.99 (0.93-1.06)	−0.11	0.915
	LPFS interp.	1.15 (1.07-1.23)	2.02	0.043	Model 2.1	LPFS interp.	1.05 (0.99-1.12)	0.78	0.438
						Dark P	0.72 (0.67-0.76)	−5.36	< 0.001
Model 3.0	mentalization	1.23 (1.16-1.31)	3.50	< 0.001	Model 3.1	mentalization	1.11 (1.05-1.18)	1.75	0.080
						Dark P	0.73 (0.69-0.78)	−4.86	< 0.001
Model 4.0	emp. concern	1.20 (1.12-1.29)	2.65	0.008	Model 4.1	emp. concern	1.11 (1.04-1.19)	1.63	0.103
	fantasy	0.98 (0.92-1.05)	−0.32	0.751		fantasy	1.00 (0.94-1.07)	0.05	0.963
	persp. taking	1.08 (1.01-1.14)	1.19	0.235		persp. taking	1.03 (0.97-1.09)	0.48	0.633
	pers. distress	0.94 (0.88-1.00)	−1.01	0.314		pers. distress	0.93 (0.87-0.99)	−1.24	0.214
						Dark P	0.72 (0.68-0.77)	−5.21	< 0.001

All variables were standardised prior being used as predictors. All models included age and gender as confounding covariates. LPFS total, total score of LPFS-BF; persp. taking, perspective taking subscale of SPF; fantasy, fantasy subscale of SPF; pers distress, personal distress subscale of SPF; emp concern, empathic concern subscale of SPF; mentalization, total score of MZQ.

The mentalisation scale (MZQ) indicated a significant propensity to choose prosocial sentences (Model 3.0). Finally, we included all IRI scales simultaneously in Model 4.0. Here, we found a significant positive association with prosocial sentences only for the IRI empathic concern scale (Model 4.0, emp. concern). All other subscales failed to reach significance.

These analyses were repeated after adding the total dark personality score to the model (**Table 2**, models 1.1 to 4.1, right columns). The effects on the personality functioning total scale (Model 1.1, LPFS total) and the LPFS subscales (Model 2.1) did not reach significance, even if the empathy/intimacy subscale LPFS interpersonal was significant in the model without adjusting for dark P scores. The effect of dark P scores, in contrast, was highly significant (Model 1.1 and 2.1, Dark P). **Figure 1A** shows the estimated density of the ratio of prosocial sentences plotted on the coordinates given by standardized dark P and empathy/intimacy scores of the subscale LPFS interpersonal. The dominant effect of dark P score is clearly visible, as the expected rate of prosocial sentences decreases almost in parallel to the increase of dark P scores on the y axis. The significant effect of this LPFS subscale in the model without adjustment appears to be due to the absence of individuals with low scores in this subscale and low dark P scores; hence, high LPFS scores are associated with lower dark P scores in the sample.

In the models adjusted for dark P score, only the mentalization scale (MZQ, Model 3.1) and the IRI empathic concern scale (Model 4.1, emp. concern) showed a positive association, but only at trend level. If corrected for multiple testing, this association would be reduced to a null finding. In contrast, in both models the effects of the dark P scores to form antisocial sentences were highly significant. **Figure 1B** shows the estimated density of the prosocial sentences ratio as a function of standardized mentalization (the most significant predictor in the model adjusted for dark P scores) and dark P scores. One can see that, at high levels of dark P scores, mentalization makes no difference to the expected rate of prosocial sentences. At low dark P scores, however, prosocial sentences increase with mentalization. As shown in the Figure, there were a few individuals with average mentalization but high dark P scores. These individuals did not induce a change in the expected rate of prosocial sentences.

The different levels of significance in the joint model of personality/empathy and dark P were reflected in the respective effect sizes. When controlling for dark P, i.e. in individuals with average dark P, an increase of one standard deviation in mentalization relative to average scores increased the expected rate of prosocial sentences from 83.0% to 84.4%, i.e. a very modest increase of 1.4%. In contrast, at average levels of personality functioning, the expected decrease in prosocial sentences due to an increase of one standard deviation in dark P scores was to 78.1%, i.e. a reduction of prosocial sentences of 4.8%. This reduction is comparable to the one found in previous larger studies of cognitions and antisocial traits (53).

In summary, our findings are consistent with the expected association between personality functioning (but specifically empathic concern and mentalization) in so far as these measures of personality functioning showed significant results in all models when adjusted for age and gender. However, when dark P scores were included as a predictor, this association disappeared. Only empathic concern and mentalisation were able to explain additional variance, but only at the trend level. Furthermore, as expected, dark P remained the strongest predictor associated with the choice of antisocial cognitions, even when controlling for interpersonal functioning or any of its associate specific scales. The **Supplementary Material** contains supplementary analyses on the role of sentence type in the association between personality traits and sentence selection that confirm these general conclusions (53).

### 3.3 Data-driven exploratory analysis

The previous analysis found only a weak trend from selected personality scales or subscales in predicting prosocial sentence choice when adjusting for dark P scores. However, it might be argued that this comparison is unfair since the sentences in the SST were explicitly developed with scales such as dark P in mind. Furthermore, the unequal success of personality scales in the association with sentence selection suggests that specific aspects of personality may be predictive of antisocial cognitions, as assessed by the SST. Personality scales and subscales might not have considered this potential association at the time of their definition. This might account for the fact that, when adjusted for dark P scores, they only reach trend significance.

To give personality scales a better chance at predicting antisocial cognitions, we conducted an exploratory analysis using an machine learning approach to identify individual items of these scales that may best predict the rate of antisocial sentences across individuals. To this end, we computed the conditional variable importance given by random forest models (RF-CVI). We first assessed the replicability of the items identified with this approach by splitting the dataset into two subsets at random and applying the analysis to these two subsets separately. We then plotted in **Figure 2A** the conditional importance of each item of these scales. This plot shows a clear association between the conditional importance computed in the two datasets, indicating the good replicability of the approach. The most important items are placed in the upper right quadrant.

Having established the robustness of this approach, we computed an RF-CVI analysis using the complete dataset. The ranking of the complete dataset is depicted in **Figure 2B**, while the highest five scoring items are listed in **Table 3**. The questions for these items are presented in **Table 3**. The initial three items primarily focus on emotional and motivational involvement, particularly towards individuals in need or suffering. The final two items address the capacity for mentalization, emphasizing that, in addition to possessing explicit ideas about the social world, understanding its meaning is essential for comprehending one’s own feelings and regulating one’s behaviour.

**Table 3:** Items selected in the RF-CVI analysis.

RF-CVI rank	Scale/item	Text
1	MZQ/5	Most of the time it is better not to feel anything
2	SP/1	I often have tender, concerned feelings for people less fortunate than me
3	SP/16	Before criticizing somebody, I try to imagine how I would feel if I were in their place.
4	MZQ/2	Explanations from others are of little assistance in understanding my feelings.
5	MZQ/4	I only believe that someone really likes me a lot if I have enough realistic proof for it (e.g., a date, a gift, or a hug).

Because the RF-CVI analysis showed two items with much larger importance than the rest, we computed a composite index obtained by averaging the standardized scores of these two items. In **Figure 2C** we visualized the distribution of antisocial sentences in the subspace spanned by this composite index and dark P. One can see that, even at high levels of dark P scores, the rate of prosocial sentences increases with the value of the composite index, in contrast to the findings depicted in **Figure 1**. The rate of prosocial sentences increases as one moves down the y axis as well as rightwards on the x axis, suggesting that the composite index and dark P contribute additively to the selection of antisocial cognitions as assessed by the SST. The same conclusion may be reached by using a composite index of the first five items in this analysis (not shown for brevity).

In the mixed effects logistic regression, this composite index was predictive of prosocial sentence selection in the SST even when adjusted for dark P scores (*z* = 4.02, *p* < 0.001). Dark P scores remained significant predictors of prosocial sentence selection (*z* = -4.22, *p* < 0.001), suggesting that the personality functioning items and dark P were separate predictors of prosocial/antisocial cognitions.

**Figure 3:**
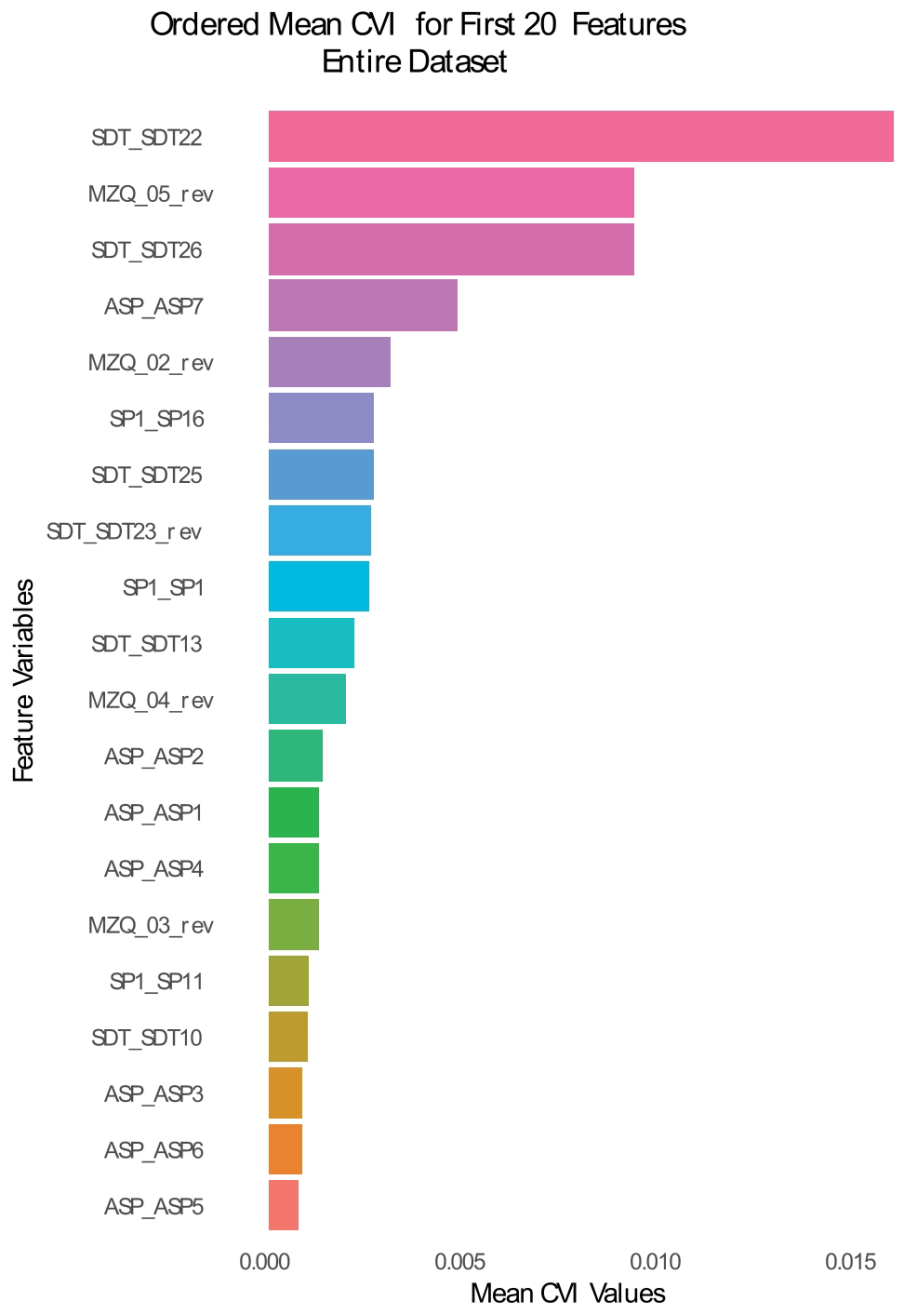
relative importance values of the first 20 highly scoring RF-CVI computed on both personality functioning and dark P items. The variables at the top are best at predicting the prosocial sentence rate across individuals.

This RF-CVI analysis was conducted without including the dark P items in the model. While intended to redress a possible disadvantage of the original personality functioning scales relative to dark P when predicting prosocial/antisocial cognitions, it may now be argued that this analysis is in turn too favourable to personality items. It may not be too surprising or indicative that items that best predict the outcome survive adjustment for dark P scores, since they were selected prior to the analysis on the basis of their effectiveness as predictors. To address this concern, we conducted a final RF-CVI analysis in which we included items from both personality functioning scales and dark P scales (**Figure 3**). This figure shows that the items selected by the first RF-CVI analysis also appear within the first items of the new analysis, interspersed with items from the dak P scales. The fifth item of the MZQ scale, in particular, appeared to be highly predictive of prosocial sentence choice even when presented together with dark P items. This confirm the suggestion of the previous analysis that selective aspects of personality functioning are additional predictors of prosocial cognitions, as assessed by the SST.

## 4 Discussion

In the present study, the propensity for antisocial cognitions, as assessed by the SST, was found to be prevalently associated with dark personality scores. There were only moderate to low associations with the LPFS interpersonal functioning scale, with the core domains empathy and intimacy, as well as the MZQ scale and the IRI empathic concern scale, and these associations disappeared almost entirely after adjusting for dark personality scores. When adjusted for dark personality, only the MZQ scale and the IRI empathic scale showed significance at a trend level. However, this finding was obtained even if dark personality scores and scales of personality functioning were themselves inversely associated, as expected. These findings are consistent with the SST capturing cognitions that are specific to antisocial traits and not for generic personality impairments. Moreover, this finding also confirms and extends that of Rabl et al. (53), where antisocial cognitions detected with the SST could be differentiated from negative cognitions associated with depressiveness and negative affect.

When examining the relative contribution of mentalization or empathy and dark personality on rates of antisocial cognitions, as detected by the SST, we found that there were some individuals with relatively high scores in both domains and that tended to entertain antisocial cognitions. While antisocial cognitions are not per se antisocial behaviour, this finding raises the possibility that some individuals with high dark personality scores possess good capacities to mentalize, contradicting a simple model in which mentalization excludes antisocial traits.

There are several mentions in the literature that individuals with antisocial traits or symptoms may possess the capacity to understand others’ thoughts and feelings, while having diminished social-affective empathic capacities that include emotional sharing, contagion, or their respective responses such as empathic concern and personal distress (74–76). For example, some studies suggest that persons high in dark personality possess at times the ability to emphasise with others (77) but do not have the disposition to do so automatically, but only when it is of their use or when they are instructed (78, 79). It has also been suggested that dark personalities demonstrate the ability to understand other people’s motives and needs in order to manipulate them for one’s own benefit without concern (39, 79, 80). Our findings are consistent with this contrastive picture: while empathy and mentalization were broadly predictive of low antisocial cognition, especially through their negative association with dark personality, there was a smaller group of individuals who departed from this pattern.

The terms empathy, theory of mind and mentalization are often used as umbrella concepts leading to confusion due to the heterogeneity of assigned various models and definitions (81). In the literature, there is an emerging consensus, suggesting at least three domains or intrapsychic processes (82, 83): the first describes the ability to share someone’s emotional experience (affective empathy), the second describes the ability to take another person’s perspective or to understand other people’s mental states (cognitive empathy) (84–91), with this latter further divided to distinguish between cognitive and affective aspects (78, 92). The cognitive component, encompassing Theory of Mind (ToM) and mentalization capacities, focuses on thoughts, wishes, and imaginations, while concurrently, the affective aspect of these capacities primarily engages with the emotional realm. Neuroimaging studies have provided support for the dissociation between affective and cognitive processes (28–30, 82–88, 92–94).

In the mentalizing literature, the proposition is put forward that the cognitive dimension aligns with mental state attribution, while the affective dimension involves shared representations (26, 31, 95–97). Both processes are considered crucial for a comprehensive clinical understanding of patients and the psychotherapeutic treatment of individuals with antisocial personality disorders (31). The complexity of the relationship between empathy (as an umbrella concept with multiple facets) and antisocial personality traits is further exemplified by the results of investigations of subgroups, such as the dark empath, revealing high antisocial tendencies accompanied by elevated empathy levels (77). Overall, the multifaceted nature of empathy and mentalization suggests that not all these facets may be equally predictive of low antisocial traits. Hence, our finding that at high antisocial personality scores mentalization capacity made no difference to the high rate of antisocial sentences is consistent with this literature. Furthermore, it illustrates why mentalization capacity had little or no predictive power for antisocial cognitions once antisocial traits were taken into account.

Beyond sharing emotional experiences and understanding mental states, a third domain of empathic function encompasses responses reflecting motivational tendencies elicited by witnessing other person´s experience of suffering, manifested as empathic concern (e.g., other-oriented feelings leading to caring behaviour) or personal distress (e.g., self-oriented feelings of discomfort leading to withdrawal) (65, 83, 98, 99). The importance of these motivational responses has been highlighted by findings that have challenged assumptions about the relationship between empathy and aggressive behaviour (100). A recent meta-analysis found no association between them (100) and criticized a potentially overly narrow conceptualization, particularly within the domain of affective empathy, that failed to capture the full complexity of the construct (100). As a result, recent literature suggests exploring the motivational tendencies of these empathic responses (100), even in contradictory emotional situations, such as taking pleasure in the pain of others. These studies too suggest that the relationship between dark personality and empathy may be more multifaceted and intricate than initially assumed.

However, our study of predictors of antisocial cognitions in the SST, which used an machine learning approach to identify single predictive items from the personality functioning scales, suggested that there were specific aspects of empathic capacities that were autonomously predictive of prosocial cognitions, i.e. over and above low dark personality traits. These items inquired about the tendency of individuals to resonate emotionally and respond, particularly with the plight of others.

Taken together, our findings suggest that prosocial and antisocial behaviours are not perfect opposites. On the one hand, we have individual with high antisocial cognitions with average to good mentalization capacity. Here, mentalization capacity is not per se predictive of antisocial cognitions. On the other hand, we have individuals with high emotional resonance to the suffering of others that produce prosocial cognitions above the rate predicted by their low antisocial scores. Empathic concern is defined as a feeling of warmth and care for others and is associated with the motivation to help, and to alleviate or prevent the suffering or pain of others (101, 102). Our finding suggests that feeling empathic concern or compassion for a person is a more relevant factor for activating prosocial schemas than sharing emotions with other people, when this construct involves a motivational component.

The partial dissociation between prosocial and antisocial behaviours may arise because the motivation to help others in need may be distinct from the motivation to take advantage of others. In terms of cognitions, this finding is analogous to that of Kienhöfer et al. (58) who presented evidence that detachment and negative affect traits were differentially associated with optimistic and pessimistic cognitions detected with an SST assessing the depressive triad, with detachment preferentially associated with optimistic and negative affect with pessimistic cognitions (103). The evaluation of potential choices involves a complex interplay of conscious and automatic components (92, 104–109), which may come into play when specific schemas are activated in the presence of motivational factors, energizing the content of cognitions. In that study, we suggested that the difference in sentence choice may be based on the distinction between motivational processes, with optimistic cognitions related to appetitive and pessimistic cognitions to aversive motivation.

From an evolutionary perspective (104–106, 110), there is a relatively large literature suggesting that cooperative, prosocial behaviour and exploitative, ruthless, antisocial behaviour are two types of strategies for interacting with conspecifics for various reasons, such as achieving goals, securing resources or feeling safe. Thus, prosocial and antisocial cognitions may underlie schemas that are not only the opposite at the cognitive level, but also manifest themselves in contrary ways at the behavioural level (e.g., caring for or harming others/feeling pleasure when others are in pain and their justifications) - what unites them is a motivation to address suffering.

## 5 Limitations

In assessing the implications and impact of our study, it is important to acknowledge and consider certain limitations that may affect the interpretation and generalisability of our findings.

First, the study relies predominantly on self-report measures for data collection. While the measurements used in the study are valuable for obtaining subjective insights, their inherent limitations, influenced by various participant-related factors, can significantly limit the objectivity and reliability of the findings.

Second, we only obtained limited variance in the dark personality scores. This might deprive important insights into understanding the underlying motivational factors, especially in combination with a high personality function.

Furthermore, the demographic composition of the sample, which is largely made up of students, is another significant limitation. This specific demographic may not be adequately representative of the wider population, particularly in terms of age, socio-economic background and cultural diversity. As a result, the generalisability of the study’s findings to a wider population may be limited.

In terms of future research directions, it is important to replicate and extend these findings in more diverse populations and different clinical settings. This would improve understanding of how these findings apply across different demographic and psychosocial backgrounds. In addition, another promising avenue for future research is to investigate how the findings from this study can be effectively integrated into therapeutic interventions for personality disorders. Exploring this could lead to more targeted and effective treatment strategies, taking advantage of the unique contributions of AI-driven analyses in clinical psychology.

## Data availability statement

The raw data supporting the conclusions of this article will be made available by the authors, without undue reservation.

## Ethics statement

The studies involving humans were approved by Board for Ethical Questions in Science of the University of Innsbruck. The studies were conducted in accordance with the local legislation and institutional requirements. The participants provided their written informed consent to participate in this study.

## Author contributions

LR: Project administration, Writing – review & editing, Writing – original draft, Funding acquisition, Formal analysis, Data curation, Conceptualization. JM: Writing – original draft, Formal analysis, Writing – review & editing. RV: Software, Project administration, Writing – review & editing, Writing – original draft, Supervision, Methodology, Funding acquisition, Formal analysis. KL: Project administration, Data curation, Writing – review & editing, Writing – original draft, Supervision, Methodology, Investigation, Funding acquisition, Formal analysis, Conceptualization.
