# Supplementary material for: The dark side of personality functioning: associations between antisocial cognitions, personality functioning (AMPD), empathy and mentalisation
Source: Front Psychiatry. 2024 May 28;15:1377177. doi: 10.3389/fpsyt.2024.1377177 (PMC11165144; doi:10.3389/fpsyt.2024.1377177)
Supplement: Supplementary file 1 [file DataSheet_1.pdf]

1 Supplementary materials

1.1 Random Forest Regression Conditional Variable Importance (RF-CVI)

We provide here details on the process we followed to fit the RF-CVI model. We began with a detailed hyperparameter optimization, employing a grid search technique that spanned a wide range of hyperparameters and assessed 720 distinct combinations. This search encompassed three critical parameters: the number of trees (ntree), which sets the count of trees to construct within the forest; mtry, determining the number of features evaluated at each node for splitting within a tree; and minsplit, specifying the minimum number of observations required for considering a split at a node.

The ranges for these parameters covered a broad spectrum of potential model configurations: ntree values were set from 200 to 1000, incremented by 100; mtry values ranged from 2 to 20, with steps of 2; and minsplit values extended from 2 to 16, also incremented by 2. This detailed and structured approach ensured a thorough examination of the parameter space, laying a solid foundation for subsequent analysis and insights into RF-CVI.

Given our primary focus on elucidating the importance of variables rather than on maximizing predictive performance, we selected an mtry of 20, a minsplit of 14, and an ntree of 1000. These parameters were applied separately to each dataset.

1.2 Supplementary analysis

The antisocial sentences SST contains sentences with two types of content: in the first type, persons in the sentence are harmed physically, humiliated, etc.; in the second, type, justificatory cognitions for self-interested or sadistic behavior are given (1). In previous studies with the SST, we found that specific aspects of dark P, such as sadism, were specifically correlated with the harm type of sentences (1).

We repeat this analysis here, looking for an interaction between types of sentences and personality traits, using for dark P the subscales (where we expect sadism to be most predictive) and the overall dark P, and the predictive personality index derived with the RF-CVI analysis.

In the first model, in which we used all dark P scales, we failed to reach significance for any interaction. However, we replicated the previous finding that sadism was most strongly associated with antisocial sentences of the harm type (rather than justification) than any other scale (sadism: $z = -1.26$, $p = 0.10$, one-tailed; Machiavellianism: $z = -0.22$, n.s., psychopathy, $z = -0.61$, n.s.; narcissism, $z = -0.23$, n.s.). The personality index gave an association with prosocial sentences (i.e. in the opposite direction) stronger in the sentences of harm than justification type ($z = 1.46$, $p = 0.07$, one-tailed).

In the second model, in which dark P was assessed by an overall score, we found a significant interaction with the type of sentences (more antisocial sentences in the harm than in the justification group, $z = -3.55$, $p < 0.001$). Again, the personality index gave a trend-level tendency to deliver more prosocial sentences in the harm than in the justification group ($z = 1.58$, $p = 0.06$, one-tailed).

In summary, this analysis confirms (1) that the harm sentences were more strongly associated with antisocial personality traits. In addition, we also found that these same sentences induced

a stronger propensity towards prosocial sentences in individuals with higher personality scores as identified by the RF-CVI analysis.

1.3 Supplementary references

1. Rabl L, Kienhöfer V, Moshagen M, Labek K, Viviani R. Exploring the dark schemas – The development of the Scrambled Sentences Task for antisocial cognitions. PsyArXiv. 2023.
